# The relationship between cognitive function and functional capacity, and cognitive reserve and reaction time in patients with multiple sclerosis

**DOI:** 10.1055/s-0044-1788273

**Published:** 2024-08-26

**Authors:** Busra Candiri, Engin Ramazanoglu, Burcu Talu, Mehmet Tecellioglu

**Affiliations:** 1 Inonu University, Faculty of Health Sciences, Physiotherapy and Rehabilitation Department, Malatya, Türkiye.; 2 Inonu University, Faculty of Medicine, Neurology Department, Malatya, Türkiye.

**Keywords:** Multiple Sclerosis, Cognition, Cognitive Reserve, Mobile Applications, Muscle Strength, Physical Functional Performance, Esclerose Múltipla, Cognição, Reserva Cognitiva, Aplicativos Móveis, Força Muscular, Desempenho Físico Funcional

## Abstract

**Background** Cognitive dysfunction is frequently seen in multiple sclerosis (MS). However, there are conflicting findings regarding the factors it is associated with.

**Objective** To investigate the relationship between aerobic capacity, strength, disability, depression, fatigue, and cognitive reserve and function.

**Methods** The mobile applications Trail Making Test (TMT A-B), Digit Span Test (DST), Visuospatial Memory Test (VSMT), and Tap Fast were used in the cognitive function evaluation. Functional performance was assessed with the 6-minute walk test (6MWT), 5-Time Sit-to-Sand (5STS) test, and grip strength. Cognitive Reserve Index (CRI), Beck Depression Inventory, Fatigue Severity Scale (FSS), and Nottingham Health Profile were also used.

**Results** A significant difference was found between the MS and control groups only in the 6MWT, STS-5, grip strength, TMT, VSMT, and Tap Fast. Good correlation was found between the TMT-A and 6MWT and physical mobility. A fair correlation was shown between grip strength, energy, and pain status. A good correlation was found between TMT-B and 6MWT, and a fair relationship with disability, cognitive reserve, and pain. Good correlation was observed between the DST and 6MWT, left grip strength, pain, and energy status; fair correlations were found between right grip strength, cognitive reserve, and physical mobility. Good correlation was found between the VSMT and energy. A fair relationship between disability, cognitive reserve, and pain was demonstrated. Good correlation was observed between the Tap Fast score and disability, 5STS, FSS, energy, and physical mobility. A fair relationship was found between pain and social isolation.

**Conclusion** It has been shown that cognitive performance in MS is related to disability, functional performance, cognitive reserve, fatigue, and general health.

**Trial registration** NCT06084182.

## Resumo

**Antecedentes** A disfunção cognitiva é frequentemente observada na esclerose múltipla (EM). No entanto, existem resultados conflitantes sobre os fatores aos quais está associada.

**Objetivo** Investigar a relação entre capacidade aeróbica, força, incapacidade, depressão, fadiga e reserva e função cognitiva.

**Métodos** Os aplicativos móveis Trail Making Test (TMT A-B), Digit Span Test (DST), Visuoespacial Memory Test (VSMT) e Tap Fast foram utilizados na avaliação da função cognitiva. O desempenho funcional foi avaliado por meio do teste de caminhada de 6 minutos (TC6), Teste de Sentar-Levantar Cinco Vezes (TSL5) e força de preensão manual. Também foram utilizados Índice de Reserva Cognitiva (IRC), Inventário de Depressão de Beck, Escala de Gravidade de Fadiga (EGF) e Perfil de Saúde de Nottingham.

**Resultados** Foi encontrada diferença significativa entre os grupos EM e controle apenas no TC6, TSL5, força de preensão, TMT, VSMT e Tap Fast. Foi encontrada boa correlação entre o TMT-A e o TC6 e a mobilidade física. Foi demonstrada uma correlação razoável entre força de preensão, energia e estado de dor. Foi encontrada uma boa correlação entre o TMT-B e o TC6, e uma relação razoável com incapacidade, reserva cognitiva e dor. Foi observada boa correlação entre o DST e o TC6, força de preensão esquerda, dor e estado energético; correlações justas foram encontradas entre força de preensão direita, reserva cognitiva e mobilidade física. Foi encontrada boa correlação entre o VSMT e a energia. Foi demonstrada uma relação justa entre incapacidade, reserva cognitiva e dor. Foi observada boa correlação entre o escore Tap Fast e incapacidade, TLS5, EGF, energia e mobilidade física. Foi encontrada uma relação justa entre dor e isolamento social.

**Conclusão** Foi demonstrado que o desempenho cognitivo na EM está relacionado com incapacidade, desempenho funcional, reserva cognitiva, fadiga e saúde geral.

**Registro de teste** NCT06084182.

## INTRODUCTION

Multiple sclerosis (MS) is an inflammatory disease of the central nervous system characterized by demyelination and axonal loss. 1 2 Frequently associated neurological symptoms include optic neuritis, gait ataxia, sensory loss, limb weakness, and cognitive dysfunction. 2 Of these, cognitive dysfunction is estimated to occur in 40 to –65% of patients with MS. Cognitive dysfunction is important for predicting disease progression, and regardless of physical disability, it causes significant limitations in daily life. 1 3 Impairments have been reported in MS, especially in information processing speed, executive function, working memory, verbal and visual memory, attention, verbal fluency, and visuospatial skills. 1 3

Individuals with similar neurological disorders may have different cognitive dimensions. This is explained by the fact that cognitive impairment is associated with many factors. 4 A study has shown that aerobic capacity and muscle strength are related to cognitive processing speed in MS. However, disability was determined to be an important moderator of this relationship. 5 A recent study found that cognitive function is associated with functional capacity and sleep. 6 Depression affects the lives of more than 20% of individuals with MS, 7 and it is a factor that contributes to the worsening of cognitive functions. 8 Fatigue is a common symptom in MS, with a prevalence of 70 to 90%. 9 A negative correlation between fatigue and cognitive function has been reported. 10 Cognitive reserve refers to the protective feature of the brain against changes caused by brain aging or neurological disorders. It has been reported that cognitive reserve has a protective effect on cognition in individuals with MS. 4 Cognitive reserve is an important factor that also affects the relationship between depression and fatigue in MS. 11

Although it appears in the literature that there are many factors related to cognitive function in MS, no research has been found that includes the effect of cognitive reserve along with functional measurements, depression, and fatigue. In this regard, the present study aims to investigate the relationship between aerobic capacity, muscle strength, disability, depression, fatigue, cognitive reserve, and cognitive function in participants with MS.

## METHODS

In this cross-sectional study, patients who presented to Turgut Ozal Medical Center Neurology Polyclinic between December 2021 and September 2022 were included. The study was approved by the Inonu University Clinical Research Ethics Committee with the decision number 2021/2611. It was conducted in accordance with the principles of the Declaration of Helsinki, and an informed consent was obtained from participants.

### Design overview

The population of study consists of patients with MS who are followed up in the outpatient treatment unit. In this unit, patients with MS are followed up on Tuesday. After the patients were attended by the specialist physician for routine medical check-ups, the neurologist encouraged them to participate in the study by giving information about the evaluation to be performed by the physiotherapist.

### Setting and participants

The study population consisted of patients who were diagnosed, by a relevant physician, with relapsing-remitting MS (RR-MS) according to the McDonald Diagnostic Criteria. In the power analysis, with α = 0.05 and 1-β (power) = 0.80, the difference between the Symbol Digit Modalities Test values of group 1 (51.7 ± 13.5) and group 2 (59.5 ± 8.9) in patients with MS was assumed to be 7.8 units. 12 It was calculated that at least 34 people should be included in each group. Sample size was calculated with Openepi version 3 ( http://www.openepi.com ). Among those who agreed to participate in the study and filled out the informed consent form, patients diagnosed with RR-MS were selected for the patient group, and the partners/spouses accompanying them were selected for the control group by the non-probability random sampling method. The inclusion criteria for the patient group were having been diagnosed with RR-MS, having an Expanded Disability Status Scale (EDSS) score < 5.5, being able to use smartphone, having no history of relapse during the 30 days prior to the study, being aged 18 to 65 years, and having a Mini-Mental State Examination score > 24. The control group included healthy individuals aged 18 to 65 years, able to use a smartphone, and without neurological or orthopedic problems.

### Questionnaires

#### *Cognitive reserve*

The Cognitive Reserve Index (CRI) was used to evaluate cognitive reserve. It consists of 20 items providing information on total years of education, duration of professional occupation and cognitive demands, leisure time activities. 13 High score means high cognitive reserve. 14

#### *Fatigue*

The fatigue severity scale (FSS) was used to evaluate fatigue. This questionnaire primarily assesses the impact of motor aspects of fatigue on activities of daily living. 1 point is scored as “strongly disagree” and 7 points as “strongly agree.” The total scoring is averaged. 15

#### *Depressive symptoms*

The Beck Depression Inventory, 2nd edition (BDI), which can be administered as a self-report, was used to evaluate depressive symptoms. 16 This questionnaire consists of 21 questions with a score between 0 and 3. The total score is 63. 17

#### *General health status*

General health was evaluated with the Nottingham Health Profile (NHP). It has 6 sub-headings, including sleep, energy, emotional reaction, physical mobility, pain, social isolation. High NHP scores indicate low health-related quality of life. 18

### Mobile applications

#### *Trail Making Test*

The Trail Making Test (TMT) is a test that provides information about many areas, such as mental flexibility, processing speed, and executive functions. The mobile application version of the test was used. In part A, numbers in the circle are marked to each other in an increasing manner. Part B contains both numbers and letters and includes the task of matching them to each other in ascending order. 19 The time to complete the tasks was recorded as a score.

#### *Digit Span Test*

A mobile application called Digit Span Test (DST) was used to evaluate working memory. The normal version of the DST consists of two subtests depending on the forward and backward increment of digits. 20 In the mobile application, the evaluation was performed in such a way that the advanced test step values increased. The score given by the application was recorded.

#### *Visuospatial Memory Test*

A mobile application called Visuospatial Memory Test (VSMT) was used to evaluate visuospatial short-term memory. Pink squares appear on the screen in the application. The squares to be marked are indicated in order. The participant is asked to make the markings in the same way. At the end of the test, the highest span scores were recorded. 21

#### *Tap Fast*

The mobile application named Tap Fast was used for the reaction time evaluation of the patients. The patient is asked to touch as many boxes as possible within 15 seconds by touching the screen as quickly as possible. The patient's score was recorded.

### Functional capacity

#### *6-Minute Walk Test*

The 6-Minute Walk Test (6MWT) was used to evaluate functional capacity. During the test, physiotherapists asked the participant to walk quickly and safely along a 40 m straight corridor. During the test, the physiotherapist walked behind the patient to prevent them from falling. The distance was recorded in meters. 22

#### *5-Time Sit-to-Stand test*

It has been shown in the literature that the 5-Time Sit-to-Stand (5STS) test is associated with lower extremity muscle strength in MS patients. The test was performed on a chair without armrests with a height of 45 cm and a depth of 41 cm. Five repetitions are performed as quickly as possible, and the test ends when the patient's back touches the chair in the last sitting. 23

#### *Handgrip strength*

Grip strength was assessed using a Baseline Digital Hand Dynamometer (300 lbs/135 kg). The test was performed with the extremity positioned according to the American Hand Therapist Association recommendations. The test was repeated three times, and the mean was recorded. 24

### Statistical analysis

The IBM SPSS Statistics for Windows, version 25.0 (IBM Corp., Armonk, NY, USA) was used to analyze the data. The normality of the data was evaluated with the Shapiro-Wilk test. The Chi-squared test was used to compare categorical variables between the groups. The independent Sample *t* -test and Mann-Whitney U-test were used to compare all continuous variables between the groups. The relationship between cognitive function and other parameters was evaluated with the Spearman and Pearson correlation tests. Correlation values ≥ 0.4 were considered satisfactory and interpreted as follows: r, 0.00 to 0.20, poor; 0.21 to 0.40, fair; 0.41 to 0.60, good; 0.61 to 0.80, very good, and 0.81 to 1.0, excellent. A *p* -value < 0.05 was considered statistically significant.

## RESULTS

**Figure 1:**
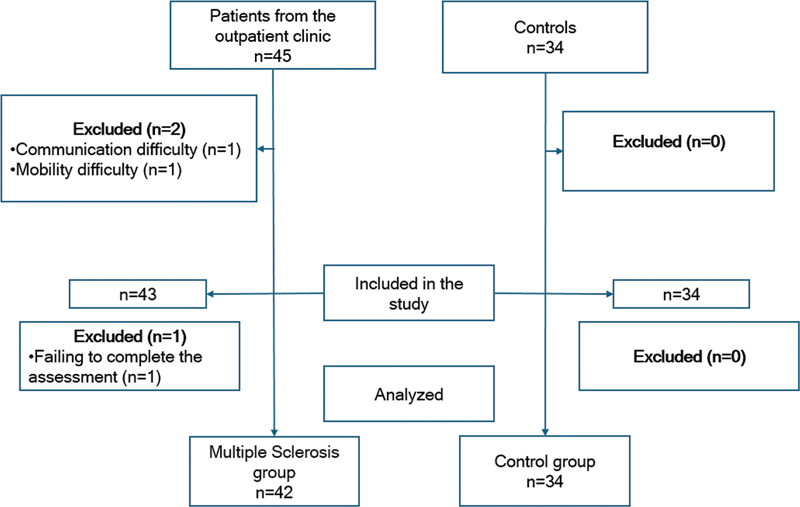
Flow diagram of the study population.

In total, 45 RR-MS patients were screened for eligibility. A total of 43 individuals were included because 2 participants were not eligible (communication and mobility difficulty). One person did not complete the evaluation. A total of 76 people in the MS and control groups were included in the analysis ( Figure 1 ). The basic demographic characteristics of the participants are listed in Table 1 .

**Table 1:** Demographic and disease characteristics of the participants in the groups

	MS ( *n* = 42)	Control ( *n* = 34)	*p*
Mean age (years)	35.69 ± 9.15	37.91 ± 11.99	0.363 ^a^
Mean BMI (kg/m ^2^ )	23.35 ± 3.94	25.31 ± 4.76	0.054 ^a^
Gender (F/M)	33/9	20/14	0.062 ^b^
Mean EDSS score	3.04 ± 0.97	–	**–**

Abbreviations: BMI, body mass index; EDSS, expanded disability status scale; F, female; M, male; MS, multiple sclerosis.

Notes: ^a^ Independent samples *t* -test (mean ± standard deviation). ^b^ Chi-squared test.

The 6MWT distance was significantly lower in the MS group than in the control group ( *p* = 0.000). The 5STS time was significantly higher in the MS group than in the control group ( *p* = 0.001). Right and left grip strength was significantly higher in favor of the control group ( *p* = 0.016; *p* = 0.004 respectively). The TMT A and B times were significantly lower in the control group than in the MS group ( *p* = 0.014; *p* = 0.000 respectively). The number of sequences in the DST was lower in the MS group than in the control group ( *p* = 0.011). There was no difference between the MS and control groups in terms of the highest sequence in the VSMT ( *p* = 0.063). The Tap Fast application score was also higher in the control group ( *p* = 0.000) ( Table 2 ).

**Table 2:** Comparison of parameters of MS and control group

	MS ( *n* = 42)	Control ( *n* = 34)	*p*
6MWT (m)	2.86 ± 0.57	3.45 ± 0.39	0.000 ^b^
STS-5 (s)	13.05 ± 2.87	11.08 ± 2.27	0.001 ^b^
Handgrip R (kg)	22.94 ± 8.27	28.07 ± 9.93	0.016 ^b^
Handgrip L (kg)	22.28 ± 7.53	28.54 ± 10.33	0.004 ^a^
TMT-A (s)	86.61 ± 39.44	71.62 ± 41.33	0.014 ^a^
TMT-B (s)	116.47 ± 45.50	80.87 ± 24.76	0.000 ^a^
Digit Span Test	5.50 ± 1.46	6.44 ± 1.50	0.011 ^a^
Visuospatial Memory Test	4.28 ± 1.46	4.76 ± 1.12	0.063 ^a^
Tap Fast	18.76 ± 5.74	23.35 ± 4.04	0.000 ^b^

Abbreviations: 5STS, Five Times Sit-to-Stand Test; 6MWT, 6-Minute Walk Test; L, left; MS, multiple sclerosis; R: right; TMT, Trail Making Test.

Notes: ^a^ Mann-Whitney U test. ^b^ Independent samples *t* -test.

The TMT-A was negatively correlated with the 6MWT (r = −0.455) and positively correlated with the NHP-physical mobility (r = 0.426). There were fair negative correlations between the TMT-A and right and left grip strength (r = −0.306; r = −0.308 respectively), and positive correlations with the NHP-pain and NHP-energy (r = 0.312 and r = 0.323 respectively). There was a good degree of negative correlation between the TMT-B and 6MWT (r = −0.401) and a fair correlation with the EDSS (r = 0.352), CRI (r = −0.327), and NHP-Pain (r = 0.308). The DST has shown a good positive correlation with the 6MWT (r = 0.446) and right grip strength (r = 0.432), and a good negative correlation with NHP-pain (r = −0.425) and NHP-energy (r = −0.401). A fair correlation was shown between the DST and CRI (r = 0.358), right grip strength (r = 0.375), and NHP-physical mobility (r = −0.341). There was a good negative correlation (r = −0.427) between the VSMT and NHP-energy. Fair correlations were found between the EDSS (r = −0.354), CRI (r = 0.358), NHP-pain (r = 0.389), and VSMT. A good degree of correlation was found between the Tap Fast and EDSS (r = −0.421), STS-5 (r = −0.412), FSS (−0.441), NHP-energy (r = −0.415), and NHP-physical mobility (r = −0.442). There was a fair correlation between NHP-pain (r = −0.307) and NHP-social isolation (r = −0.323) ( Table 3 ).

**Table 3:** Correlation of cognitive function and other parameters

		TMT-A ^a^	TMT-B ^a^	Digit Span Test ^a^	Visuospatial Memory Test ^a^	Tap Fast
BMI (kg/m ^2^ )	r	0.105	0.106	−0.093	−0.303	−0.132
	*p*	0.507	0.505	0.558	0.051	0.405 ^b^
EDSS score	r	0.220	0.352*	−0.292	− 0.354*	− 0.421**
	*p*	0.162	**0.022**	0.061	**0.021**	** 0.005 ^a^**
6MWT (m)	r	− 0.455**	− 0.401**	0.446**	−0.273	0.188
	*p*	**0.002**	**0.009**	**0.003**	0.081	0.233 ^b^
STS-5 (s)	r	0.179	0.225	−0.261	0.232	− 0.412**
	*p*	0.256	0.152	0.095	0.140	** 0.007 ^b^**
Handgrip R (kg)	r	− 0.306*	−0.198	**0.375**	0.226	0.257
	*p*	**0.049**	0.210	**0.014**	0.151	0.101 ^b^
Handgrip L (kg)	r	− 0.308*	−0.205	0.432**	0.236	0.172
	*p*	**0.048**	0.193	**0.004**	0.132	0.277 ^a^
CRI	r	−0.212	− 0.327*	0.358*	0.376*	0.181
	*p*	0.177	**0.034**	**0.020**	**0.014**	0.252 ^b^
BDI	r	0.161	0.141	−0.270	−0.248	−0.141
	*p*	0.307	0.373	0.084	0.114	0.372 ^a^
FSS	r	0.282	0.198	−0.270	−0.184	− 0.441**
	*p*	0.070	0.209	0.084	0.244	** 0.003 ^b^**
Nottingham Health Profile						
Energy	r	0.323*	0.245	− 0.425**	− 0.427**	− 0.415**
	*p*	**0.037**	0.118	**0.005**	**0.005**	** 0.006 ^a^**
Pain	r	0.312*	0.308*	− 0.401**	− 0.389*	− 0.307*
	*p*	**0.044**	**0.047**	**0.009**	**0.011**	** 0.048 ^a^**
Emotional reactions	r	0.213	0.127	−0.290	−0.178	−0.154
	*p*	0.176	0.423	0.062	0.259	0.330 ^a^
Sleep	r	0.102	0.225	−0.192	−0.073	−0.239
	*p*	0.519	0.152	0.223	0.646	0.128 ^a^
Social isolation	r	0.207	0.150	−0.229	−0.084	− 0.323*
	*p*	0.187	0.343	0.145	0.598	** 0.037 ^a^**
Physical mobility	r	0.426**	0.264	− 0.341*	−0.283	− 0.442**
	*p*	**0.005**	0.091	**0.027**	0.069	** 0.003 ^a^**

Abbreviations: 5STS, Five Times Sit-to-Stand Test; 6MWT, 6-Minute Walk Test; BDI, Beck Depression Inventory; BMI, body mass index; CRI, cognitive reserve index; EDSS, Expanded Disability Status Scale; FSS, Fatigue Severity Scale; L, left; R, right; TMT, Trail Making Test.

Notes: ^a^ Spearman correlation test. ^b^ Pearson correlation test. * *p* < 0.05. ** *p* < 0.01.

## DISCUSSION

In the present study, cognitive function and functional parameters were shown to be negatively affected in participants with MS. Additionally, cognitive function in these patients was found to be associated with disability, 6MWT, cognitive reserve, grip strength, energy, pain, social isolation, and physical mobility.

In our findings, supporting the literature, it was shown that functional capacity, lower extremity muscle strength, and grip strength were negatively affected. 25 In general, participants with MS perform worse on cognitive tests than healthy individuals. 26 Thinking that mobile applications would provide significant convenience in the clinic, we chose to use freely accessible mobile applications for cognitive function. In our results, there was an impairment in TMT and DST performance compared with the control group. Impairments in attention, memory, verbal fluency, visuospatial memory, and general cognitive function were reported in the group of RR-MS patients, compared with healthy control group. 27 Supporting our findings, the current study showed significantly higher completion times in the TMT-A and B compared with the control group. 28 However, interestingly, although there was a lower span in visuospatial perception than the control group, no significant difference was found between the groups. In evaluation 10 years later, deterioration in other areas of cognitive function occurred before deterioration in visuospatial perception. 29 Another study showed that the impairment is greater in individuals with MS who have visual complaints. 30 We did not record disease duration in our study. In addition, while serious visual impairment was an exclusion criterion, we did not question vision-related problems in detail. These situations may explain the lack of significant differences in our results. Additionally, the delay in reaction time in the MS group is consistent with the literature. 31

There was no relationship between the body mass index (BMI) and the parameters of cognitive function in this sample. In one study, only the association between BMI and attention was preserved in RR-MS after controlling for gender, age, and EDSS. 32 Contrarily, in other studies, BMI and body fat percentage and whole body fat ratio did not cause a significant relationship with cognitive function in MS. 33 34 The complexity of the results may be related to the assessment of different aspects of cognitive function in the studies and the heterogeneous study group regarding different types of MS. Severe disability (EDSS > 4) is important in interpreting the relationship between physical fitness and cognitive functions because it affects mobility. 35 The fact that EDSS mean in this sample was 3.04 ± 0.97 indicates that the relationship may be significant. There was a relationship between EDSS score and TMT-B, visuospatial perception, and reaction time. Our findings demonstrate an association between higher disability and worse cognitive performance.

While a relationship was shown between cognitive processing speed and aerobic capacity and lower extremity muscle strength in participants with mild MS, there was no significant relationship with verbal and visual memory measures. 5 In another study, physical functions in MS patients had a significant relationship only with information processing speed. 12 In our results, which support the literature, 6MWT had a significant relationship with other cognitive function measurements, except visuospatial perception and reaction time; and grip strength had a significant relationship with TMT-A and DST. Interestingly, STS-5 had a significant relationship only with reaction time. In addition, there was significantly worse performance than the control group. This may be related to the domains of cognitive function that we assessed. Therefore, our results are important for understanding the relationship between cognitive flexibility, working memory and visuospatial perception, and lower extremity muscle strength.

The literature mostly focuses on the protective role of cognitive reserve on cognitive functions.A study by Tremblay et al. has shown that cognitive reserve is important in the relationship between brain matter volume and cognition. 4 In a study by Artemiadis et al., it was found that cognitive reserve was significantly related to information processing speed, verbal memory, and visual-spatial memory. 36 In our findings, there was a relationship with visual-spatial memory, supporting the literature. Additionally, working memory and cognitive flexibility were also significantly related.

A study by Yigit et al. had findings similar to ours, that depression did not have an effect on cognitive functions. 37 In addition, it has been stated that depression is more related to processing speed, executive functions, attention, and memory, while anxiety is more related to visual-spatial memory and verbal learning abilities. 38 Accordingly, our focus on depression and our evaluation of cognitive function in a limited area such as processing speed, working memory, visuospatial memory and reaction time may have affected our results.

A recent study by Guillemin et al. has shown that fatigue is an important determinant of cognitive functions in MS. 38 In the study by Yigit et al., fatigue was only related to processing speed. 37 However, in both of these studies, there were different types of MS. It has also been stated that fatigue has a multidimensional structure and different interactions with cognitive functions. 38 In the present study, no significant relationship was found between fatigue and cognitive functions. The fact that our sample consisted only of RR-MS and that we did not distinguish between physical and cognitive fatigue may have affected our results. However, the lack of a relationship between sleep, social isolation, and emotional reaction sections of general health and cognitive performance also supports the lack of a relationship between cognitive performance and depression and fatigue. In our findings, there were significant relationships between cognitive function and energy, pain and physical mobility. In a recent study, physical health related to quality of life was significantly associated with cognitive function. 39

One of the strengths of the study is that we used mobile applications to evaluate cognitive function. Additionally, this study is the first to investigate the relationship between cognitive function and disability, functional parameters, cognitive reserve, depression, fatigue and general health in individuals with MS and to compare these parameters with a healthy control group. However, there are some limitations. Evaluating cognitive functions in a limited area to benefit from mobile applications may have reduced the generalizability of our data. Our limited sample size is also another reason. It would be useful to evaluate different dimensions of cognitive function in larger samples in future studies.

In conclusion, we found that the functional performance and cognitive functions of individuals with RR-MS were negatively affected compared with the control group. Cognitive function has been shown to correlate with disability, functional capacity, cognitive reserve, fatigue, and general health. In addition, no relationship was shown between cognitive function and body mass index, depression, and emotional reactions related to general health and sleep.
